# Implicit vs. Explicit Emotion Processing in Autism Spectrum Disorders: An Opinion on the Role of the Cerebellum

**DOI:** 10.3389/fpsyg.2020.00096

**Published:** 2020-01-31

**Authors:** Libera Siciliano, Silvia Clausi

**Affiliations:** ^1^PhD Program in Behavioral Neuroscience, “Sapienza” University of Rome, Rome, Italy; ^2^Ataxia Laboratory, IRCCS Fondazione Santa Lucia, Rome, Italy; ^3^Department of Psychology, Sapienza University of Rome, Rome, Italy

**Keywords:** cerebellum, emotions, autism, implicit processing, explicit processing

## Introduction

Human beings are continuously exposed to external and internal emotional stimuli which are processed to create adaptive social relations. Based on the nature of emotional phenomena and on the effort required for their elaboration, emotional processing could be viewed along a continuum, ranging from an implicit/unconscious level to an explicit/conscious level (Lane, 2000). The implicit processing of emotions is meant to be an automatic, procedural, and non-conceptual process that does not require conscious access to be executed. Instead, explicit processing requires declarative evaluation and involves higher cognitive resources to define conscious emotional states (Damasio, 1994; Lane, 2000).

Within the extended neural network involved in the emotional field, the implicit processing of emotions has been linked to the amygdala and the anterior cingulated cortex (Critchley et al., 2005; Webb et al., 2010), while the temporoparietal junction and the medial prefrontal cortex have been reported to be mainly involved when the conscious attribution of emotions and intentions is required (Saxe and Kanwisher, 2003; Saxe and Powell, 2006).

However, the neural networks underpinning these processes are still far from being well-defined (Schaller and Rauh, 2017).

Over the years, many scientific studies have recognized the cerebellum as being part of these brain networks (Stoodley and Schmahmann, 2010; Baumann and Mattingley, 2012; Leggio and Olivito, 2018; Clausi et al., 2019a; Van Overwalle et al., 2019a) and have evidenced its role in affective and emotional functioning (Adamaszek et al., 2015; Clausi et al., 2015, 2019b; Lupo et al., 2015, 2018). Accordingly, functional and anatomical connections have been found between the cerebellum and the cortical and subcortical structures involved in both implicit and explicit emotional processing (Critchley et al., 2000; Singer et al., 2004; Schutter et al., 2009; Stoodley and Schmahmann, 2010; Schraa-Tam et al., 2012).

The contribution of the cerebellum to implicit and explicit mechanisms underlying emotion processing has been recently acknowledged (Clausi et al., 2017).

Indeed, the cerebellum is involved in implicit aspects such as the modulation of autonomic reactions, the automatic component of emotional learning associated, e.g., with fear conditioning (Critchley et al., 2000; Sacchetti et al., 2005; Timmann et al., 2010), and in the implicit processing of emotional facial expression (Schutter et al., 2009; Clausi et al., 2017). The cerebellar vermis might play a role in this stage of emotion elaboration through its connections with the brainstem (catecholamine neurons), the hypothalamus (Snider and Maiti, 1976), and the limbic areas, such as the amygdala and hippocampus (Sacchetti et al., 2005).

Instead, regarding the explicit aspects, the cerebellum is involved in the self-perception of negative emotions and in the integration of internal state information with external environmental stimuli to consciously and adaptively elaborate emotions (Timmann et al., 2010; Clausi et al., 2017, 2019a).

The posterior portions of the cerebellum might contribute to these aspects by means of their connections with cortical areas involved in more complex features of emotional evaluation (i.e., the medial prefrontal cortex and temporoparietal junction) (Rudebeck et al., 2008; Buckner et al., 2011).

Evidence about the “emotional cerebellum” has also been conveyed by clinical studies. Indeed, a pattern of emotional and affective disorders has been found in patients with cerebellar damage as part of the well-known cerebellar cognitive affective syndrome (Schmahmann and Sherman, 1998; Tavano et al., 2007).

Intriguingly, converging clinical and neuroimaging evidence points to a cerebellar involvement in the emotional disturbances and social cognition impairments described in people with autism spectrum disorders (ASD) (Fatemi et al., 2012; D'Mello and Stoodley, 2015).

ASD are clinically complex and heterogeneous neurodevelopmental conditions characterized by core impairments in social interaction, repetitive behaviors, and restricted interests (American Psychiatric Association, 2013; Goldson, 2016). Emotional processing is a challenge for people with ASD, whose impairments have been reported for both implicit and explicit components (Senju, 2013; Lozier et al., 2014; Kana et al., 2016).

Nevertheless, these aspects are often separately investigated, leading to controversial conclusions (Ben-Shalom et al., 2006). Indeed, while most of the literature on people with ASD agrees on the existence of implicit emotional processing impairment, i.e., when the task requires the elaboration of emotional facial expression (Baron-Cohen et al., 1997, 2001), controversial results emerge when the task requires the explicit elaboration of emotions. In the latest case, the heterogeneous nature of the ASD condition and camouflaging phenomena due to learned compensatory strategies might lead to the fallacious assumption that people with high-functioning ASD (hf-ASD) do not show such deficits (Senju, 2012; Schuwerk et al., 2014; Schaller and Rauh, 2017). For example, this could happen in tasks in which context information can help the person explicitly attribute emotions to others (Frith and Frith, 2012; Senju, 2012; Schaller and Rauh, 2017). However, it must be considered that when the amount of context information increases, the environment becomes more confusing, and the compensation ability may no longer guarantee the processing of emotional information (Frith and Frith, 2012). Indeed, when the complexity increases, the person has to integrate emotional processing with mentalizing ability to correctly infer emotional states (Mier et al., 2010). This complex social elaboration is impaired in people with hf-ASD when dynamic video-based stimuli close to everyday life are used or when various aspects of social situations are analyzed (Dziobek et al., 2006; Schaller and Rauh, 2011, 2017). Thus, the heterogeneity of capacities shown in a complex condition such as ASD, together with the variety of tasks used in different studies, may lead to controversial results.

The complex behavioral outcome of people with ASD has been linked to the functional alteration of complex neural circuits encompassing several brain areas, such as the parietal, temporal, and frontal regions (Abell et al., 1999; Carper et al., 2002; Hazlett et al., 2006; Minshew and Williams, 2007), as well as subcortical structures (Sparks et al., 2002; Amaral et al., 2008; Cauda et al., 2011). Among these, the cerebellum has been consistently recognized as part of the distributed neural networks affected in people with ASD (Wang et al., 2014; D'Mello and Stoodley, 2015; Olivito et al., 2017; Stoodley et al., 2017). Indeed, evidence of structural and functional alterations in specific cerebellar regions and in cerebello-cortical networks underlying emotional processing has been reported in this population (Courchesne et al., 1988; Khan et al., 2015; Stoodley et al., 2017; Arnold Anteraper et al., 2019). In particular, a decrease in the Purkinje cell number in the cerebellar vermis and a gray matter reduction in the posterior cerebellum have been found in people with ASD (Ritvo et al., 1986; Bauman and Kemper, 2005; Fatemi et al., 2012), together with altered functional connectivity between the posterior cerebellum and the frontal and temporal areas involved in mentalizing abilities (Olivito et al., 2017).

In this framework, considering the suggested connections between the cerebellum and the cortical and subcortical structures involved in implicit and explicit emotional processing (Schutter et al., 2009; Stoodley and Schmahmann, 2010; Clausi et al., 2017) and the association between the cerebellum and the social-emotional impairments in ASD (Wang et al., 2014; D'Mello and Stoodley, 2015), in the present opinion, we will provide some novel insights into the possible nature of implicit and explicit emotion processing deficits in ASD related to cerebellar-specific involvement. To this aim, we will take into account the most primitive ability to process facial emotions to analyze the implicit emotional process and the capacity to integrate intricate environmental information with the theory of mind abilities to explicitly infer complex emotional states.

## Cerebellar Implicit Emotional Processing in Autism

One of the most primitive and implicitly learned processes crucial for social interactions is the ability to elaborate emotions conveyed by facial expressions (Schaller and Rauh, 2017). This ability and that of spontaneous emotional mimicry are part of the implicit processes that contribute to emotional contagion (McIntosh et al., 2006; Senju, 2013). An adaptive phenomenon linked to the implicit processing and learning of emotions is the habituation to recurrently presented faces, as measured by the time spent looking at them (Webb et al., 2010). This ability allows individuals to face predictable social stimuli in an automatic way, since facial expressions convey emotional cues that human beings typically process first when they are embedded in social situations (Shyman, 2017). In typically developing children, this phenomenon is associated with decreased neural responsiveness in the amygdala when repeated facial stimuli are presented (Webb et al., 2010).

Interplay between the cerebellum and the amygdala has been found to contribute to the process of implicit emotional learning (Snider and Maiti, 1976; Zhu et al., 2011). Animal studies have shown that a bidirectional interaction between the basolateral amygdala and the cerebellum allows learning-related plasticity in fearful conditions by increasing the firing in vermal Purkinje cells, thus inducing learning-related long-term potentiation in the cerebellum (Snider and Maiti, 1976; Zhu et al., 2011). In this way, the cerebellum integrates sensory and emotional information, enabling appropriate reactions to new fearful situations and maintaining them across time and contexts (Zhu et al., 2011). Accordingly, in fear conditioning paradigms, animal and human studies have shown an involvement of the cerebellar vermis in the associative mechanisms that contribute to the creation of memory traces with emotional valence (Sacchetti et al., 2002; Labrenz et al., 2015). Because of its extensive connections with limbic areas, the cerebellar vermis has been defined as the “limbic cerebellum” (Schmahmann, 1991).

Within this framework, it is important to emphasize that the automatization and implicit processing of emotional mechanisms require the repetitive and predictable patterns of stimuli.

Interestingly, the correct recognition of spatial and temporal relations among relevant actions has been associated with cerebellar predictive computing in motor and non-motor domains (Leggio et al., 2008; Molinari et al., 2008). Furthermore, cortical plastic changes mediated by cerebellar-driven facilitation have been described when a predictable pattern is conveyed by the current stimuli (Molinari et al., 2002; Ito, 2005).

Supporting cerebellar involvement in the emotional domain, clinical studies have revealed that damage in the cerebellar vermis is associated with autism-like behaviors in cerebellar patients, giving rise to difficulties in the automatic attribution of relevant emotional states regardless of the context, as in emotion recognition from facial expressions (Schmahmann and Sherman, 1998; Riva and Giorgi, 2000).

The elaboration of facial emotional expression and automatic emotional mimicry are reported to be the earliest social impairments in people with ASD (Baron-Cohen et al., 2000; McIntosh et al., 2006; Senju, 2013). These alterations are often related to dysfunction of the mirror neurons system (MNS), which is a set of brain regions active in both action execution and the observation of actions performed by others (Press et al., 2010).

Remarkably, it has been found that children with ASD show a slower habituation to recurrently presented faces compared to typically developing children (Webb et al., 2010). This diminished habituation has been correlated with social symptoms (Swartz et al., 2013). Consistently, in people with ASD, repeated exposure to faces, conveying both positive and negative emotions, has not been associated with decreased activity in the amygdala (Swartz et al., 2013; Tam et al., 2017).

Moreover, in these people, structural imaging studies have shown gray matter abnormalities in the vermis and hypoplasia of this cerebellar portion due to cellular defects in the Purkinje cells in early life (Courchesne et al., 2011; Fatemi et al., 2012; Wang et al., 2014).

We posit that, in people with ASD, cerebellar dysfunction and an altered interaction between the cerebellum and the amygdala might affect the implicit process of emotion elaboration, thus impeding habituation. Our assumption is that the alteration of the cerebellar vermis might prevent the detection of operative internal models for spatially and temporally organized emotional phenomena, thus impeding automatic responses for incoming emotional stimuli and their prediction and automated implementation when facing analogous events in the future (Leggio and Molinari, 2015).

## Cerebellar Explicit Emotional Processing in Autism

The explicit processing of emotions requires subjects to interpret several idiosyncratic and environmental stimuli and needs a detailed appraisal based on the interplay between present and past states (Schaller and Rauh, 2017).

When emotional stimuli (i.e., facial expressions) are embedded in complex social situations, the concomitant processing of mental states may be required (Schaller and Rauh, 2017).

In this case, the capacity to infer others' emotional states is closely related to the attribution of their intentions and requires a declarative and a higher cognitive evaluation (Brothers and Ring, 1990).

The brain network supporting explicit emotional processing involves the cortico-pulvinar-cortical pathway (i.e., the medial frontal cortex, the superior temporal gyrus, and the cingulate cortex) and the supra-modal association cortices belonging to the default mode network (DMN) (Grimm et al., 2008; Sreenivas et al., 2012; Shobe, 2014).

It is well-known that the posterior cerebellar hemispheres, named the neocerebellum, play a role in high associative functions in synchrony with co-evoluted regions of the cerebral cortex recruited, for example, when more cognitive aspects of emotional processing are in demand (Adamaszek et al., 2017; Leggio and Olivito, 2018). Consistently, these cerebellar portions have been found to be functionally connected to cerebral association areas belonging to the DMN. In healthy subjects, fMRI studies have shown that these cerebello-cortical networks are involved in social-emotional tasks that require high-order cognitive reasoning (Buckner et al., 2011; Van Overwalle et al., 2015).

Furthermore, reduced functional connectivity (FC) between cerebellar posterior lobules, such as the right Crus-II, and cortical regions involved in complex social-emotional reasoning is present in patients with cerebellar degenerative atrophy, whose behavioral profile is characterized by mentalizing impairment and the inability to deduce emotional states (Clausi et al., 2019a).

As described above, studies on the ability to explicitly process emotions in people with ASD have yielded controversial conclusions (Senju, 2012; Schaller and Rauh, 2017). Indeed, some studies have shown that people with hf-ASD succeed in simple explicit tests in which it is required to attribute emotions to protagonists of social stories and where additional context information helps to deduce emotional states (Schaller and Rauh, 2017). However, when the context complexity is higher and people with hf-ASD are required to integrate facial emotion expressions, intentions and emotional states, their performance becomes worse (Schaller and Rauh, 2011, 2017).

The preserved processing and recognition of basic emotions in people with hf-ASD has usually been associated with compensatory strategies gained by declarative mechanisms, thanks to spared cognitive and executive functions and to “environmental scaffolding” built on external resources (Frith and Frith, 2012). Indeed, people with hf-ASD show typical outcomes in explicit tests when overtly instructed to attribute emotions to protagonists of social stories (Frith and Frith, 2012). Therefore, the recruitment of declarative/explicit strategies might support compensation in neurodevelopmental disorders and could explain the variability in symptom severity described in hf-ASD (Ullman and Pullman, 2015; Livingston and Happé, 2017). However, it has been speculated that the environment may either facilitate or inhibit compensation (Livingston and Happé, 2017), since growing environmental demands may exceed the compensatory ability (Schaller and Rauh, 2017).

Relatedly, when the complexity and novelty of a social context increases, the compensation of people with hf-ASD less efficiently overcomes their difficulties in emotion processing (Frith and Frith, 2012).

It has been proposed that underconnectivity between brain areas, including those of the DMN, could be the neural basis of the socioemotional impairment in ASD, as asserted by the theory of the underconnected brain in ASD (Just et al., 2007; Müller, 2007; Müller et al., 2011).

Moreover, among the various theories, the hypothesis of developmental diaschisis assumes that cerebellar dysfunction during critical periods could disrupt the development of far neocortical networks, including the aforementioned supra-modal associative cortices (Lai et al., 2014; Wang et al., 2014). The disruption of the fronto-cerebellar network across developmental stages may give rise to motor, emotional and social symptoms (Igelström et al., 2017), mainly associated with a general impairment of the predictive process (Sinha et al., 2014). Sinha et al. (2014) argued that prevented access to predictive processes impedes people with ASD from using previously learned relationships between events and stimuli, thus being constrained to interpret behaviors based only on basic environmental sensory signals.

Interestingly, fMRI studies on people with ASD have shown abnormal FC between regions of the DMN comprising the temporoparietal junction and the medial prefrontal cortex and posterior lobules of the cerebellum, such as Crus-I/II, involved in the mentalizing process (D'Mello and Stoodley, 2015; Olivito et al., 2018). Thus, we hypothesize that atypical interplay between these lobules and supra-modal cortices might impede explicit emotional processing when context complexity increases and that further social effort is required for the conscious attribution of emotions and intentions (Saxe and Powell, 2006).

## The Cerebellar Prediction in Emotional Processing

The cerebellum is known to act by generating operative internal models of spatially and temporally organized events comparing individuals' external events with their internal state (Ito, 2008; Molinari et al., 2008). These internal models allow us to predict incoming events and to modulate responses implicitly (Leggio and Molinari, 2015). Indeed, in the presence of cerebellar damage, the required rapid and continuous exchange of information between the internal model and the external stimuli might not be operative, thus interfering with the automatic processes (Clausi et al., 2019a).

According to the cerebellar sequence detection model, the detection and simulation of a sequence of events can either occur implicitly through experience or deliberately through overt effort (Leggio and Molinari, 2015). Indeed, analogous to information processing in the sensorimotor domain, the cerebellum might modulate high-order cortical activity (Middleton and Strick, 2001) by detecting predictable sequences of emotionally salient events based on internal models previously encoded and by allowing optimized feedforward control (Heleven et al., 2019; Van Overwalle et al., 2019b). When social novelty and environmental demands increase, explicit emotional processing may be supported by such a cerebellar operational mode; i.e., the cerebellum may exert a continuous checking on the accordance between the anticipated event based on social and emotional information and the ongoing behavior by means of projections from the cerebellar posterior regions to the areas of the cerebral cortex involved in high-order social behavior (Ito, 2008; Van Overwalle et al., 2019b). In this way, the cerebellum supports more sophisticated forms of prediction and guarantees fluid control of social-emotional processing and interactions (Clausi et al., 2019a; Van Overwalle et al., 2019b). Altogether, this mechanism allows for the regulation and adjustment of future emotional expectations and guarantees adaptive social behaviors.

As outlined in the previous section, Sinha et al. (2014) hypothesized the impairment of the predictive process as the central thread of core behavioral problems in ASD and suggested a role of the cerebellum among the key brain areas implicated in the prediction.

In this framework, we posit that in people with ASD, a dysfunction of the phylogenetically older portion of the cerebellum (i.e., the vermis), combined with its abnormal interaction with the amygdala, may impede the creation and detection of internal models for predictable and elementary social-affective signs, which are needed to process emotional stimuli automatically (Lozier et al., 2014; Wang et al., 2014). This dysfunction would prevent the correct attribution of emotional valence to upcoming social stimuli in a non-conceptual and advantageous way, thus not providing people with ASD with permanent and successful social-emotional implicit mechanisms (e.g., ability to elaborate emotions conveyed by facial expressions).

In addition, we hypothesize that when people with hf-ASD are required to explicitly process basic and “primitive” emotions, they would still be able to use their compensatory strategies. However, when social novelty and environmental demands increase, people with hf-ASD cannot benefit from cerebellar predictive control, which is crucial to adapting the previously learned responses across the context. Indeed, since the posterior cerebellum is implicated in the generation of internal models of social-emotional interactions, altered functional connectivity within the Crus-II–DMN network may affect the ability of people with hf-ASD to predict others' emotional states when the social demands require extending the encoded internal models to ambiguous or new contexts in a flexible way. This might result in difficulties engaging in adaptive social relations, typically entailing complex dynamics requiring the integration of multimodal emotional and mentalizing information.

## Conclusion and Future Investigations

Neuroimaging data on ASD agree on the presence of both structural and functional alterations involving the cerebellar vermis and the more posterior part of the cerebellum (Crus I/II). These regions are known to be active when an implicit processing of emotional stimuli is required, thus contributing to emotional memory formation, and when the explicit interpretation of emotional states requires complex inferences and concomitant mentalizing processes.

**Figure 1:**
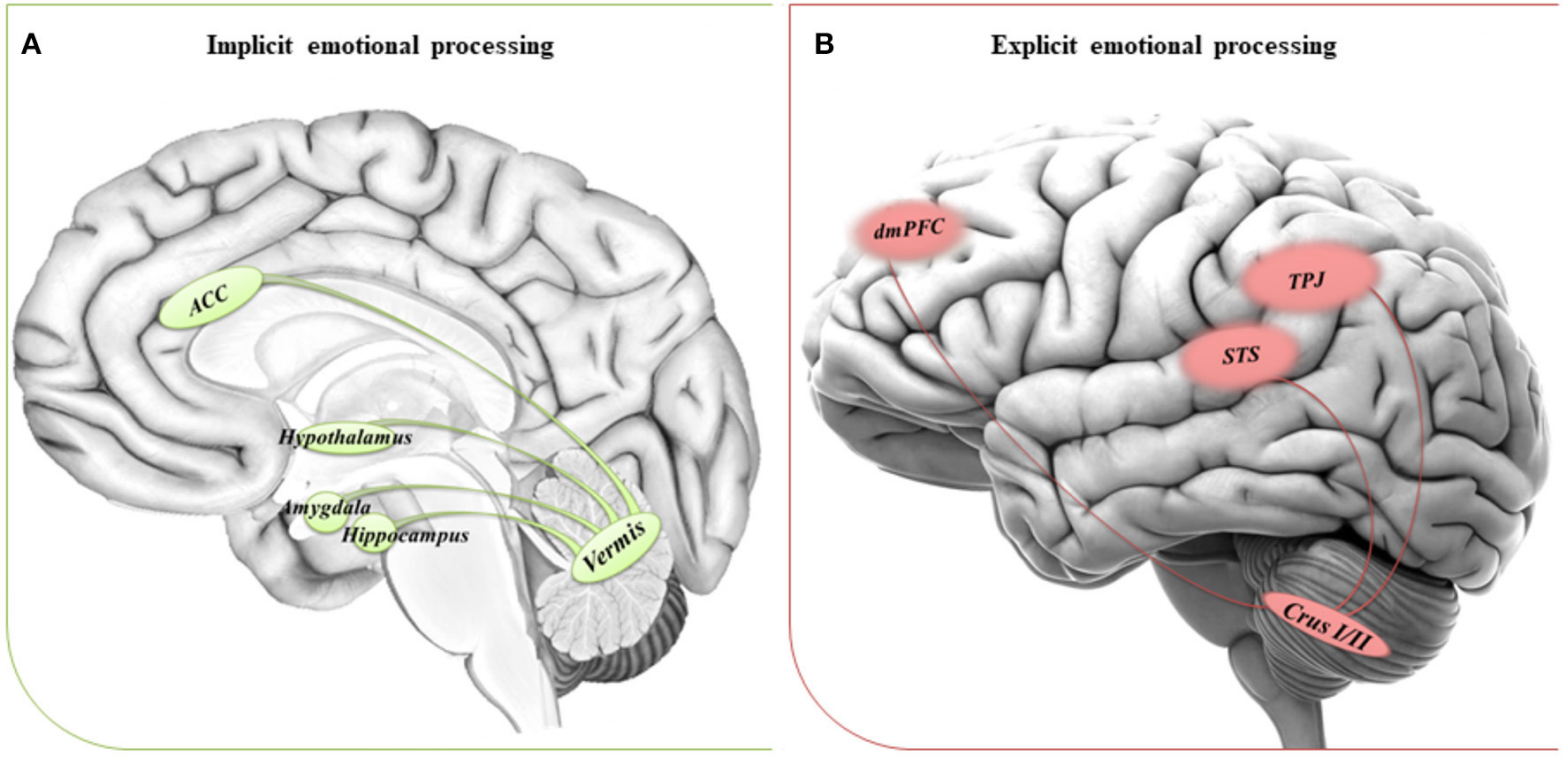
Suggested networks for implicit and explicit emotional processing. **(A)** The circuitry involved in implicit processing of emotions comprises the cerebellar vermis and the anterior cingulate cortex (ACC), hypothalamus and areas of the limbic system, such as amygdala, and hippocampus. **(B)** The circuitry involved in explicit processing of emotions comprises the cerebellar posterior lobules Crus I/II and cerebral areas of the DMN, such as the dorsomedial prefrontal cortex (dmPFC), the temporoparietal junction (TPJ), and the superior temporal sulcus (STS).

We suggest that the impaired ability to implicitly process emotions in people with ASD could be due to a dysfunctional circuitry involving the vermis and the limbic system. On the other hand, a dysfunctional reorganization of the network comprising the CrusI/II and cortical regions involved in complex social reasoning may account for the inability to compensate for the difficulties in explicit tasks when the environmental complexity increases the emotional processing demands. See Figure 1 for a schematic view of the cerebral-cerebellar networks proposed.

Conclusively, the underlying mechanisms of both implicit and explicit emotional impairments may be due to a dysfunctional cerebellar modulation on specific cerebral areas, as supported by the theory of the cerebellar operational mode described above. It is worth noting that since the cerebellum is anatomically and functionally connected to many of the cortical areas taking part in the MNS and the mentalizing network (Strick et al., 2009; Buckner et al., 2011; Van Overwalle et al., 2019a,b), it is conceivable that the hypothesis we developed for implicit and explicit emotional processing may be suitable for explaining the impairment in the mirroring system and in higher mentalizing abilities in people with ASD.

Overall, our theoretical hypothesis constitutes a framework for a new perspective on the role of the cerebellum in emotional processing dysfunctions in people with ASD.

Further experimental research is needed to better define the nature of cerebellar involvement in the socioemotional domain because it could shed further light on the pathogenesis of ASD and provide innovative insights into novel therapeutic interventions and neuromodulation targets. To this end, recognizing that the increasing complexity and novelty of contexts and stimuli could impact performance in experimental designs and everyday life, we suggest a stronger distinction between the investigation methods for implicit and explicit emotion processing. Finally, comparisons between behavioral and neuroimaging data across different pathologies with both cerebellar and emotional dysfunctions will be crucial to confirm or reject our assumption.

## Author Contributions

LS: writing and preparation of manuscript. SC: supervising as expert on the topic and critical review of manuscript. All authors listed have contributed to the concept, design, revision, and approval of manuscript.

### Conflict of Interest

The authors declare that the research was conducted in the absence of any commercial or financial relationships that could be construed as a potential conflict of interest.
